# An experience of vascular access for hemodialysis in Brazil

**DOI:** 10.1186/1755-7682-4-16

**Published:** 2011-05-15

**Authors:** Guilherme Centofanti, Eliane Y Fujii, Rafael N Cavalcante, Edgar Bortolini, Luiz Carlos de Abreu, Vitor E Valenti, Adilson C Pires, Hugo Macedo Jr, Yumiko R Yamazaki, Soraya G Audi, José R Cisternas, João R Breda, Valdelias X Pereira, Edson N Fujiki, João A Correa

**Affiliations:** 1 Departamento de Cirurgia, Departamento de Morfologia e Fisiologia, Faculdade de Medicina do ABC, Santo André, SP, Brasil; 2 Laboratório de Escrita Científica, Departamento de Morfologia e Fisiologia, Faculdade de Medicina do ABC, Santo André, SP, Brasil; 3 Departamento de Patologia, Faculdade de Medicina, Universidade de São Paulo, São Paulo, SP, Brasil

## Abstract

**Background:**

The analysis of hemodialysis services is relevant for the quality of life of patient. In this study we investigated the profile of vascular access used for hemodialysis patients in our Unit.

**Methods:**

We evaluated 219 patients of both genders aged over 18 years old who have undergone implant or manufacture of vascular hemodialysis access. We excluded patients on renal replacement therapy by peritoneal dialysis.

**Results:**

Associated diseases were hypertension and diabetes mellitus. 161 had arteriovenous fistula, with 153 held by the same dialysis and nine of them were still maturing. 27 patients on dialysis used central venous catheter. 148 were indigenous and five were made using polytetrafluoroethylene prosthesis (PTFE). Among the 27 patients with central venous catheters, ten used short-term catheter and 17 used long-term catheter. The most frequent type of fistula use was on the radio distal cephalic, in 85 patients (52.5%), followed by radio cephalic proximal in 26 patients (16%). The number of fistulas in dialysis patients conducted by this kind of therapy ranged from one to ten and in 64 patients (41.83%) fistula was the first and only to be made. Among the fistula for dialysis patients, the highest prevalence was radio cephalic fistula in 111 patients (72.5%) and mean duration of use was 48.1 months, ranging from two months to 17 years.

**Conclusion:**

Our Unit of hemodialysis is above the limits established by international norms.

## Background

The need for a vascular access is as old as hemodialysis and its adequate functioning is essential for effective maintenance dialysis [1-5]. The ideal access allows a safe approach, provides sufficient flow to perform hemodialysis and has a low complication rate [1,6,7]. Among the main access, through native fistula, fistula with prosthesis and central venous catheters, a native fistula comes closest to these premises [1,5-7]. Guidelines from different countries recommend its use [2,6-9] and studies showed that the native access presents the best patency (4 to 5 years) and lower rate of reoperation when compared with other accesses [6,7].

Catheters are associated with high rates of infection and may compromise the subsequent manufacture of fistulas [10,11]. Complications of vascular access are the main causes of morbidity in chronic renal dialysis patients and contribute to a high percentage of hospitalizations, resulting in high treatment costs [1,6,7,12]. As a consequence, in recent years it has been emerging worldwide consensuses that aim to standardize the use of access in order to reduce complications and to promote greater longevity of the fistula and improve the patient's quality of life [2].

The NKF-DOQI (National Kidney Foundation - Dialysis Outcomes Quality Initiative) - Clinical Practice Guidelines for Vascular Access, published in 1997 and its updates, is an American consensus that establishes guidelines and strategies for their implementation in order to increase the rate of preparation of native fistulas, aiming patient identification which is evolving with kidney failure and protection of local fistulas production. After its achievement, the dialysis units must implement a program to detect accesses at risk, complication rates and implement procedures to maximize accesses longevity [1,6,7].

Therefore, the analysis of frequent dialysis services is essential for its adequacy in relation to recommended guidelines and also to improvements of vascular access with reduced morbidity and improved quality of life of dialysis patients [1,2]. This context prompted us to conduct this research, aiming to monitor the quality of service and maintain control over the goals advocated by these institutions. Thus, this study was undertaken to evaluate the profile of vascular access used for hemodialysis in patients from our Unit.

## Method

### Population

This is a descriptive transversal observational study conducted on April to May 2008. We performed the study on the dialysis unit in the Department of Nephrology of the Faculdade de Medicina do ABC, in the Padre Anchieta Teaching Hospital and in the Mario Covas Hospital. The sample consisted of 219 patients (103 males). The study was approved by the Ethics Committee of the Faculty of Medicine of ABC, with questionnaires and physical examination in all patients on dialysis. All patients gave informed consent. All procedures were in compliance with the Helsinki Declaration.

### Inclusion and exclusion criteria

We included patients of both genders aged over 18 years old, which were in agreement with the consent term, who have undergone implant or manufacture of vascular hemodialysis access. We excluded patients on renal replacement therapy by peritoneal dialysis.

### Variables

We evaluated the following variables: gender, age, time which the subject was using hemodyalisis, comorbidity, actual and previous access and access complication.

### Statistical Analysis

For the descriptive statistics we used the Microsoft Excel^® ^program.

## Results

We investigated a total of 180 patients, 99 (55%) from Padre Anchieta Hospital and 81 (45%) from the Mário Covas Hospital. Regarding gender, 98 patients were males and 82 females. The average age was 52.32 years old, ranging from 18 to 92 years old. The most common etiology was renal hypertension, which was found in 40.5% of patients. Associated diseases were hypertension and diabetes mellitus (Table 1).

**Table 1:** Distribution of patients according to associated diseases.


***Associated diseases***	***Number of patients***	***%***

***Hypertension***	154	86
***Diabetes mellitus***	53	30
***Neoplasia***	0	0
***Coronary failure***	01	0.5
***Systemic Lupus Erythematosus***	05	2.8
***Others***	06	3.2

Among the patients, 161 had arteriovenous fistula, with 153 held by the same dialysis and nine of them were still maturing. 27 patients on dialysis used central venous catheter (Table 2). Among the 153 patients who underwent hemodialysis through arteriovenous fistula, 148 were indigenous and five were made using polytetrafluoroethylene prosthesis (PTFE) (Table 2). Among the 27 patients with central venous catheters, ten used short-term catheter and 17 used long-term catheter (Table 2).

**Table 2:** Distribution of vascular accesses in use.


***Type of vascular access***		***Number***	***%***

**Arteriovenous fistula**	**Native**	148	82.2
	**PTFE**	05	2.8
**Catheter**	Short-term*	10	5.5
	Long-term**	17	9.4
**Total**		180	100

%: percentage.

* Five patients with maturing fistula.

** One patient with maturing fistula.

PTFE: polytetrafluoroethylene.

Considering the patients with short-term catheter and without maturing fistula (six patients), the average time of using was 32.3 days (minimum of seven days and up to 60 days). Patients on dialysis for long-term catheter and without maturing fistula (12 patients) had a mean time of 5.46 months of use and had already exhausted their chances of making fistulas. The most frequent type of fistula use was on the radio distal cephalic, in 85 patients (52.5%), followed by radio cephalic proximal in 26 patients (16%) (Table 3).

**Table 3:** Distribution of fistulas according to location.


***Type of fistula***	***Number***	***%***

***Distal radio cephalic***	85	52.5
***Proximal radio cephalic***	26	16
***Brachial cephalic***	25	15
***Brachial basilic***	12	7.5
***PTFE upper limb***	04	2.5
***Sapheno-femoral***	01	0.6
***Total***	149	100

PTFE: polytetrafluoroethylene.

The number of fistulas in dialysis patients conducted by this kind of therapy ranged from one to ten and in 64 patients (41.83%) fistula was the first and only to be made. Among the fistula for dialysis patients, the highest prevalence was radio cephalic fistula in 111 patients (72.5%) and mean duration of use was 48.1 months, ranging from two months to 17 years. These patients had an average of 0.89 prior fistulas, and in 46 patients (30%) it was the only fistula. Among the most frequent complications observed in fistulas in use, the pseudo-aneurysm after puncture and venous hypertension were the most common.

## Discussion

It was created in 1997, the NKF-DOQI, establishing guidelines for standardization of care for chronic kidney illness to dialysis in relation to vascular access in order to decrease the complications and cost, improve the dialysis quality, thus, improving the patients quality of life, since it was reported high number of use and complications of vascular access for hemodialysis due to catheter use and fistula with prosthesis [13]. The periodic review of access for hemodialysis is intended to be performed on all services in order to monitor their adequacy in relation to international guidelines. According to these premises, we performed at the Unit of Nephrology from our University an investigation which aims to verify the adequacy and monitor the vascular access, within the standards established by the guidelines (NKF-DOQI 2006).

The number of catheters reported in our study is in accordance with the recommended by the NKF-DOQI and a large proportion of patients with short-term catheters presented fistulas in maturation. Making fistulas in patients before dialysis is a target in our service, it is difficult because our patients are often at the end-stage of renal disease. Patients with long-term catheters are at the stage of exhaustion of vascular accesses and some of them had fistulas at maturity which is also in agreement with the guidelines.

In a recent investigation published by our group, which evaluated saphenofemoral fistula as vascular access for hemodialysis [3], it was observed some cases of thrombosis due to prolonged arterial hypotension and one case due to trauma of the fistula site at home environment. By studying this type of complication, we alert to the importance of maintaining regular surveillance with periodic evaluation of these fistulas in order to detect early dysfunction so that it may be corrected in time, reducing the risk of thrombosis and increasing the usefulness period of the fistula [14].

According to our findings the percentage of patients under dialysis with arteriovenous fistulas was higher than the recommended by the NKF-DOQI 2006 (70%). Furthermore, the location of fistulas is in accordance with the guidelines. We reported predominance of distal radio-cephalic, which has a high rate in the primary fistulas, which is the ideal combination for the patient because it is related to a lower complication rate and, hence, improved quality of life [6,7].

Some issues should be addressed when taking vascular access for hemodialysis. According to a recent study, the limitation of saphenofemoral arteriovenous fistula as hemodialysis access is given in cases when the patient presents saphenous vein absence or when the saphenous vein is inadequate for this purpose and also in patients with arterial occlusive disease in the femoropopliteal territory. Another limitation of this technique is that the saphenous vein prevents the development of the fistula due to its developed muscle layer, similar to the cephalic vein in the internal forearm arteriovenous fistula. Although it prevents aneurysmal dilatation it increases the risk of myointimal hyperplasia after repeated punctures of the arteriovenous fistula [15]. On the other hand, because it is autologous material, it presents low cost, higher infection resistance and it is easy to handling, the advantages compensate its limitations even when compared to other access techniques in lower limbs which also uses autologous material such as transposition of the superficial femoral vein, first described by Huber et al [16], which reported two cases of use of this vein, one in the thigh and one in the arm and also reported by Gradman et al [17], in a retrospective study of 25 cases, which used this technique in lower limbs. This technique, which is an exception procedure, showed very good results in its long-term use according to our findings.

In addition, other complications such as distal ischemia, venous hypertension, cardiac decompensation, anastomotic pseudoaneurysm, aneurysmal dilatation and infection may be observed. For instance, Taylor et al [18] performed 45 grafts ("in loop" and "in thigh"), in whom polytetrafluoroethylene prosthesis were used in 39 cases and bovine carotid artery in six cases. They observed high rate of non-thrombotic complications with 18% of infection and 16% of distal limb ischemia.

Our findings are of great relevance because the complications with vascular access, especially catheters, are major causes of morbidity and mortality in dialysis patients [1,6,7,12]. Therefore, our findings fit with the guidelines recommendation [6,7], which is important for improving quality of life of patients with renal dialysis.

## Conclusion

The Unit of hemodialysis from our University is above the limits established by international norms, as evidenced by the analysis phase of the study.
